# Ecological and Biological Studies of Two Larval Parasitoids on Two *Monochamus* Vectors of the Pinewood Nematode in South Korea

**DOI:** 10.3390/insects15120943

**Published:** 2024-11-29

**Authors:** Moo-Sung Kim, Il-Kwon Kim

**Affiliations:** Korea National Arboretum, Pocheon-si 11186, Gyeonggi-do, Republic of Korea; cindy5138@korea.kr

**Keywords:** *Cyanopterus flavator*, *Spathius verustus*, *Monochamus*, biological control agent, pine wilt disease, pinewood nematode

## Abstract

Pine wilt disease, caused by the pinewood nematode, affects *Pinus densiflora*, *P. koraiensis* Siebold and Zucc., and *P. thunbergii* Parl. in South Korea. It is primarily spread by vectors such as *Monochamus alternatus* Hope and *M. saltuarius* (Gebler). However, biological control research in this context remains limited in South Korea. We identified 15 parasitoids of these vectors, namely, two ectoparasitic larval parasitoids, *Cyanopterus flavator* (Fabricius) and *Spathius verustus* Chao, both in Braconidae, Hymenoptera. A field survey was conducted in Pohang from 2018 to 2020 to elucidate the ecological and biological characteristics of these parasitoids. The average parasitism rates of *C. flavator* were 6.3% and 1.0%, whereas those of *S. verustus* were 0.7% and 0.5% on *M. alternatus* and *M. saltuarius*, respectively. Both *C. flavator* and *S. verustus* appeared to prefer *M. alternatus* to *M. saltuarius*, but a statistically significant difference was observed only for *C. flavator* (*p* < 0.001). The peak season and tree height with the highest parasitism rate of *C. flavator* coincided with the period when *M. alternatus* larvae lived beneath the bark and the highest larval density of *M. alternatus* and *M. saltuarius* occurred in pine trees. These findings provide valuable information for determining the optimal release time and height for *C. flavator* to achieve effective biological control.

## Simple Summary

The present study aims to search for potential biological agents on two longhorned beetle species, namely, *Monochamus alternatus* and *M*. *saltuarius*, that are known as insect vectors of the Pinewood nematode, causing the pine wilt disease in pine trees in South Korea. Potential parasitoids can be used for biological control against those vectors to slow down the spread of the nematode. The outdoor surveys were conducted from 2018 to 2020 in a southern area where uncountable pine trees were severely damaged and killed by the nematode. As a result, we identified 15 parasitic wasps in total and selected two dominant parasitic wasps, namely, *Cyanopterus flavator* and *Spathius verustus*. Both wasps attacked the larvae of both *Monochamus* vectors, but they showed a tendency to attack *M. alternatus* more. Overall, *C*. *flavator* appears more advantageous as a biological control agent than *S*. *verustus* due to having a higher parasitism rate, a stronger preference to *M*. *alternatus*, and an occurrence time synchronized with the early larval period of *M. alternatus*.

## 1. Introduction

Pine wilt disease originated in the United States and has spread to East Asia (Korea, Japan, Chinese mainland, and Taiwan), Mexico, and Europe (Portugal, and Spain) [1,2,3,4,5,6,7]. It was first detected in South Korea in October 1988 on Mt. Geumjeong in Busan and has since spread across the country [8,9]. Pinewood nematode (*Bursaphelenchus xylophilus* Steiner and Buhrer, 1943) is a causative agent that infects hosts such as red pine (*P. densiflora*), Korean pine (*P. koraiensis* Siebold and Zucc.), and black pine *(P. thunbergii* Parl.) [10]. The nematode reproduces quickly in the sapwood and blocks the movement of water and nutrients from the roots, thereby causing rapid tree death [11,12,13].

The pinewood nematode, measuring approximately 1 mm, needs a vector for transmission because it lacks mobility and mainly uses species from the genus *Monochamus* in Cerambycidae. Of the 122 *Monochamus* species reported globally, 13 are confirmed vectors of pine wilt disease [14]. In South Korea, *Monochamus alternatus* Hope, 1842 and *M. saltuarius* Gebler, 1830 have been reported as vectors [15].

In South Korea, the natural enemies of these vectors include four parasite wasp species (*Dolichomitus cephalotes*, *D. curticornis, Sclerodermus harmandi,* and *Spathius verustus*) and one predatory beetle species (*Dastarcus helophoroides*) [16,17,18]. Kim et al. performed field surveys using sentinel logs outdoors and documented the ecology and parasitism rates of an ectoparasitic braconid species [18]. However, compared to physical and chemical control studies, biological control research remains limited in South Korea, underscoring the need for this study.

A 3-year field study (2018–2020) was conducted in Pohang, a severely affected area, to identify effective biological control agents for population control of *M. alternatus* and *M. saltuarius*. We aimed to investigate (1) the parasitoid communities that prey on pine wilt disease vectors; (2) the biological characteristics of dominant parasitoid species, such as occurrence timing, parasitism rates, host preferences, and the preferred developmental stages of the hosts; and (3) the spatial distribution of dominant parasitoid species within forest stands.

## 2. Materials and Methods

### 2.1. General Status of the Study Site

**Figure 1:**
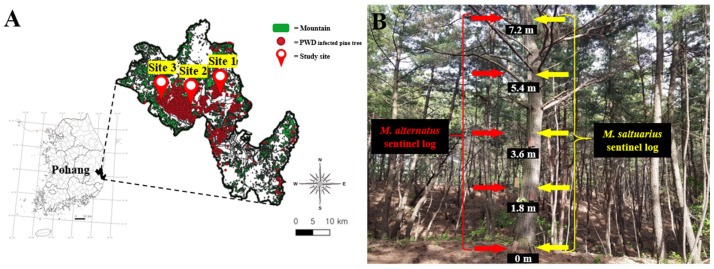
Study sites and installation methods of sentinel log. (**A**) Location of survey sites in Pohang, Gyeongsangbuk-do, South Korea; (**B**) sentinel logs of *M. alternatus* and *M. saltuarius* installed at 0 m, 1.8 m, 3.6 m, 5.4 m, and 7.2 m from the ground.

Pohang was classified as a severely affected area for pine wilt disease in 2012, following the first occurrence in 2004 [19]. Both *M. alternatus* and *M. saltuarius* are present in this region [15,20]. We selected three survey locations in Pohang (Figure 1A); all these sites comprise pine forests that have been impacted by pine wilt disease. The average diameter at breast height (DBH) of conifers, average tree age, and crown closure of pine forest in the study area ranged from 18 to 28 cm, 40 to 50 years, and 0.7 to 1.0, respectively.

### 2.2. Production of Sentinel Logs

To effectively search for the natural enemies of *M. alternatus* and *M. saltuarius*, sentinel logs were prepared indoors and then exposed in the field [21]. *P. koraiensis* was chosen as the host tree because the *M. saltuarius* larva has a much higher survival rate on *P. koraiensis*, and *M. alternatus* shows a preference for oviposition on *P. koraiensis* over other conifers [22,23]. Sentinel logs were installed to facilitate oviposition by adult *M. alternatus* and *M. saltuarius* in captivity. *P*. *koraiensis* that were not infected by wood boring beetle through visual inspection were prepared and cut into uniform-size blocks (15 cm wide, 25 cm long, and 5 cm thick); precautions were taken to handle them carefully by minimizing the usage of forestry machinery, such as skidders, knuckleboom loaders, or shovel loggers, and moving them by hand to prevent the bark from falling off. In 30 plastic breeding boxes (60 cm long × 40 cm wide × 28 cm high), three pieces of *P. koraiensis* logs and 10 pairs of *M. alternatus* or *M. saltuarius* adults, along with fresh pine branches as food, were placed to induce oviposition for 1 week. The sentinel logs, which received eggs over the week (*M. alternatus*: average 7.1 larvae, *M. saltuarius*: average 8.6 larvae), were kept indoors for a week considering the developmental period of the eggs of pine wilt disease vectors [24] to facilitate the search for larval parasitoids. Before placing them outdoors, each log was numbered on the back, and holes were drilled at the top for hanging ropes.

### 2.3. Installation of Sentinel Logs for Parasitoid Survey

We conducted a field survey for 3 years, from 2018 to 2020, using sentinel logs to search for parasitoids and determine the parasitism rates. To evaluate differences across forest depths, we strategically installed sentinel logs on three pine trees, spaced 20 m apart, starting from the forest edge and moving inward. Additionally, to compare parasitism rates at different tree heights, sentinel logs were positioned along the tree trunk from ground level (0 m) up to 7.2 m at intervals of 1.8 m (Figure 1B). The installation involved using a slingshot mechanism: a sentinel log was hoisted with a 5 mm thick rope attached to a sandbag weighing approximately 340 g. The sandbag was then launched over the tree canopy using a long-pole slingshot (240 cm) to attach to a branch. Once in position, the logs were attached with a 2 mm diameter wire to the rope and then adjusted to the desired height by pulling the rope from the opposite side.

To examine the timing of parasitoid occurrence, we installed sentinel logs at 2-week intervals from the second week of May to the fifth week of September, resulting in a total of 10 installations for each year. This schedule was designed to align with the activity periods of *M. alternatus* and *M. saltuarius* in South Korea according to previous studies [25,26]. For each installation, 45 sentinel logs were deployed for each *Monochamus* species (three sites × three trees × five sentinel logs per tree), totaling 2700 sentinel logs during the study from 2018 to 2020 (90 logs per installation × 10 installations per year × three years).

### 2.4. Identification and Observation of Developmental Stages of Parasitoids and Survey of the Emergence Period of Vectors

After 2 weeks of field exposure, the logs were retrieved and brought back to the laboratory. A small, sturdy pocketknife was used to remove the bark carefully without damaging the host larvae or any parasitoids, at whichever stages they might be. When parasitoids were found, both the host and parasitoids were gently transferred to small plastic cups (4.5 cm diameter, 30 mL capacity) and reared at an ambient temperature (25 ± 1 °C) with a relative humidity of 50 ± 5% until they matured into adults. The number of host larvae and parasitized larvae as well as details of the sentinel log installations were documented.

Changes in the developmental stages of the parasitoids feeding on host larvae were observed and recorded daily. Dead wasp specimens were prepared as dry samples and stored alongside the remains of the parasitized host larvae, with particular attention given to preserving the head capsules. All the specimens obtained from this study are now preserved in the Entomological Collection of the Korea National Arboretum. The developmental stages of the parasitoids were examined using a stereo microscope (Leica M205A Stereozoom microscope, Leica, Microsystems, Solms, Germany) and photographed with a Leica DFC 495 camera mounted on the microscope. Images were composited using LAS software (version 4.1.0, Leica Microsystems, Balgach, Switzerland) and, subsequently, edited with Adobe Photoshop CS6 (Adobe Systems Incorporated, San Jose, CA, USA). Samples of different parasitoids were first classified using the relevant taxonomic literature—Braconidae following Petersen-Silva et al. [27], Chalcidoidea according to the study of Gibson et al. [28], Bethylidae referring to Lim et al. [29], and Ichneumonidae according to the study by Broad [30].

The width of the head capsules of dead host larvae was used to determine the preferred larval instars of host larvae for parasitoids. The specific larval instars were identified based on Go et al. [31] and Kojima and Katagiri [32] for *M. alternatus* and Fan et al. [33] for *M. saltuarius*.

For density analysis of *M. alternatus* and *M. saltuarius* at each study site, we installed a single funnel pheromone trap (2-(undecyloxy) ethanol, Happy sol/(Inc.) Turf, Osan, Gyeonggi, Republic of Korea) using a rope from April to August, considering their activity periods. The pheromone trap installed at each site was exposed for 4 months without replacement of the pheromone, which lasts 3 to 4 months as instructed by the product company, and the number of captured adults was recorded biweekly [20].

### 2.5. Preliminary Survey for Selection of Dominant Biological Control Agents

Before conducting the main experiment, a preliminary study was conducted in 2018 to select useful biological control agents for the vectors. Using sentinel logs at the study site, all parasitoids of *M. alternatus* and *M. saltuarius* were surveyed. The parasitoid’s dominance was determined based on its parasitism rate.

### 2.6. Data Analysis

A likelihood ratio test was performed to compare the parasitism rates of parasitoids across the different host species with the lme4 package in R [34,35]. The interaction effect analysis was used to test for an interaction between the height of sentinel logs and forest depth. We also examined the influence of environmental variables on the parasitism rate of parasitoids using a generalized linear mixed model (GLMM) implemented with the lme4 package in R [35]. The GLMM was chosen due to the indication that the distribution of parasitism was binomial [36]. In the data of the present study, the target variable is the parasitism rate; fixed effects are the height of sentinel logs and forest depth, and the random effects are site and year. A *t*-test was used to compare the head size of parasitized larvae based on sex of the parasitoid. Statistical analyses, including likelihood ratio tests, GLMM, and *t*-test, were performed using R version 4.2.1 [37].

## 3. Results

### 3.1. Dominant Parasitoids Species of M. alternatus and M. saltuarius

From the field experiments conducted at three study sites in Buk-gu, Pohang, Gyeongsangbuk-do, South Korea, a total of 15 species of parasitoids were identified from the larvae of the two *Monochamus* species (Figure S1): (1) seven parasitoid species on *M. alternatus* larvae consisting of four Braconidae species (*Spathius verustus* (Chao, 1977), *Doryctes striatellus* (Nees, 1834), *Cyanopterus flavator* (Fabricius, 1793), and *Rhaconotus formosanus* (Watanabe, 1934)) and three Ichneumonidae species (*Xorides sepulchralis* (Holmgren, 1860) and Ichneumonidae sp. 1 and sp. 2) and (2) 11 parasitoid species on *M. saltuarius* larvae consisting of four Braconidae species (*C. flavator*, *S. verustus*, *D. striatellus*, and *R. formosanus*), one Bethylidae species (*Sclerodermus harmandi* (Buysson, 1903)), and seven Pteromalidae species (*Heydenia* sp. 1–2 and Pteromalidae sp. 1–5).

The four Braconidae species (*C. flavator*, *S. verustus*, *D. striatellus*, and *R. formosanus*) parasitized both *M. alternatus* and *M. saltuarius* larvae. Among them, *C. flavator* exhibited the highest parasitism rate for both *M. alternatus* and *M. saltuarius* at 5.8% and 1.6%, respectively. Among the others, while *S. verustus* ranked second in parasitism rate for *M. alternatus* (1.3%), it was the third for *M. saltuarius* at 0.5%, with *D. strialtellus* ranking second at 0.6%. Given the substantial difference in parasitism rates between *S. verustus* and *D. strialtellus* (1.3% vs. 0.2%) in *M. alternatus, C. flavator* and *S. verustus* were identified as the dominant species (Table 1).

### 3.2. Biological Characteristics of Parasitoids

This study newly identified *C. flavator* as an idiobiont ectoparasitic and solitary parasitoid of *M. alternatus* and *M. saltuarius* (Cerambycidae). The young larvae of *C. flavator*, observed during the disassembly of sentinel logs, were transparent white, and they attached to the exterior of the host larvae to feed on the larval hemolymph. The fully mature larvae, measuring approximately 5–6 mm in length, developed a white fatty substance in approximately two thirds of their body just before spinning a cocoon inside the hostcreated tunnel. They use a fine thread emitted from their mouth to cover themselves. The pupae were creamy white with indistinct features, but as development progressed, the head and thorax turned black while the abdomen became yellow. The pupae measured approximately 6–7 mm in length. The pupal period lasted for approximately 6–7 d at room temperature (25 ± 3 °C), during which the host larvae completely desiccated, leaving only the head part. In contrast, *S. verustus*, an ectoparasitic wasp on *M. alternatus* and *M. saltuarius* larvae, is a gregarious parasitoid rather than a solitary one. It forms cocoons in the host-created tunnel and undergoes a similar pupal period at room temperature (25 ± 3 °C). However, the fully mature larvae of *S. verustus* were smaller, measuring only 2–3 mm in size.

The total parasitism rates of *C. flavator* on *M. alternatus* and *M. saltuarius* were 6.3% and 1.0%, respectively, indicating a statistically significant preference for *M. alternatus* (*p* < 0.001) (Table 2). The sex ratio (males/males + females) of *C. flavator* was 0.8 on *M. alternatus* and 0.9 on *M. saltuarius*; the wasp produced more males on both hosts (Table 2). The total parasitism rates of *S. verustus* on *M. alternatus* and *M. saltuarius* were 0.7% and 0.5%, respectively, showing a slightly higher preference for *M. alternatus* but this was not statistically significant (Table 2). The sex ratio of *S. verustus* on both hosts was 0.2 on *M. saltuarius* and 0.2 on *M. alternatus*, resulting in the production of more females (Table 2). The overall parasitism rates across both *Monochamus* vectors were 3.4% for *C. flavator* and 0.6% for *S. verustus*, indicating a substantially higher parasitism rate for *C. flavator*.

The widths of the head capsules of host larvae parasitized by *C. flavator* and *S. verustus* were measured to determine the host larval instars and the corresponding parasitism rates were analyzed. *C. flavator* parasitized the first to eighth instar larvae of *M. alternatus*, predominantly targeting the fourth instar larvae (51.7%). For *M. saltuarius*, it parasitized the first to four instar larvae, with the highest preference for the second instar larvae (57.1%) (Table 3). *S. verustus* parasitized the second to fifth instar larvae of *M. alternatus*, mainly the fourth instar larvae (50.0%), and the first to third larvae of *M. saltuarius*, showing a notable preference for the first instar larvae (59.1%) (Table 3).

It was tested whether the gender of *C. flavator* progenies was influenced by *M. alternatus* larval instars, indicated by the host size herein. There was no statistical difference in the larval head capsule between parasitized *M. alternatus* larvae at varying instars yielding male and female offspring parasitoids (Table 4).

### 3.3. Parasitism Rates of C. flavator and S. verustus According to Tree Height and Forest Depth

The average parasitism rate of *C. flavator* significantly (*p* < 0.001) increased with height, peaking at 5.2% at 7.2 m (Table 5). Conversely, the parasitism rates by *C. flavator* did not vary significantly (*p* < 0.001) with forest depth, recording 3.4% at the forest edge (0 m) and 3.6% and 3.3% at 20 m and 40 m inside from the edge, respectively (Table 5).

The interaction effect between sentinel log height and forest depth was not significant. As opposed to *C. flavator*, the highest average parasitism rates of *S. verustus* were at 1.8 m (1.0%), followed by 5.4 m (0.7%), and the height-dependent variation in the average parasitism rates of *S. verustus* was statistically significant (*p* < 0.01) (Table 5). The parasitism rates of *S. verustus* showed non-significant variation across different forest depths, recording 0.6% at the forest edge (0 m), 0.5% at 20 m inside, and 0.6% at 40 m inside, and there were no statistically significant differences among these depths (Table 5).

### 3.4. Seasonality in the Parasitism Rates of C. flavator and S. verustus

**Figure 2:**
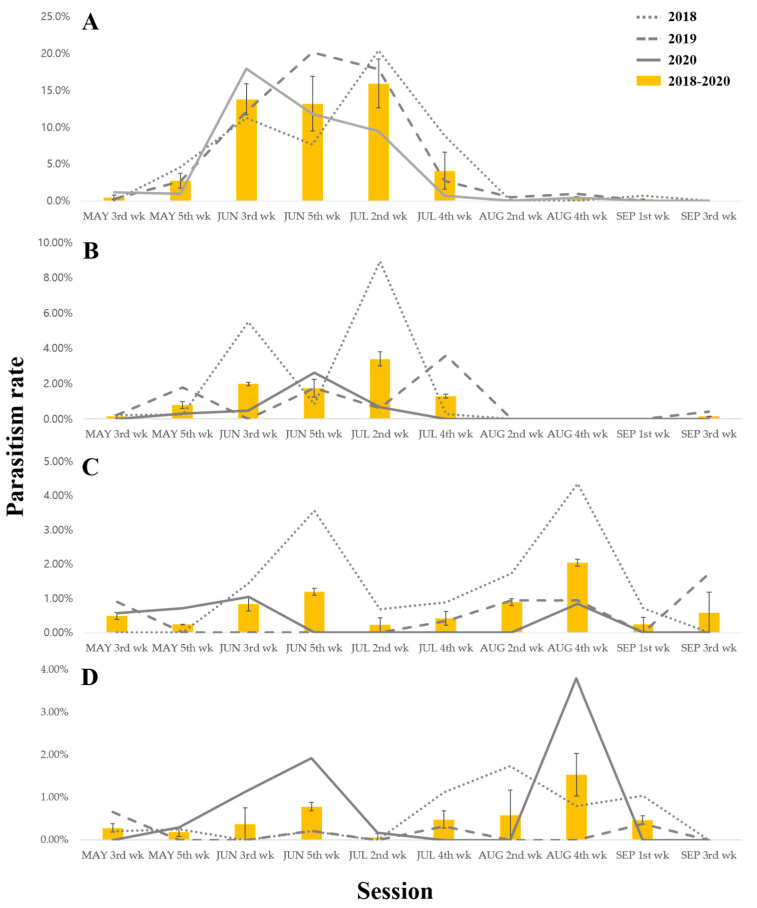
Parasitism rate fluctuation across the season by *C. flavator* and *S. verustus*. *(***A**) Relationship between *C. flavator* and *M. alternatus*. (**B**) Relationship between *C. flavator* and *M. saltuarius*. (**C**) Relationship between *S. verustus* and *M. alternatus*. (**D**) Relationship between *S. verustus* and *M. saltuarius*.

For 3 years, we surveyed the average seasonal parasitism rates of two parasitoid species. The average seasonal parasitism rate of *C. flavator* in *M. alternatus* began at 0.5% ± 0.3% in the third week of April and increased to 2.7% ± 1.0% by the fifth week of May. The average seasonal parasitism reached peak rates of 13.8% ± 2.1%, 13.2% ± 3.7%, and 15.9% ± 3.3% in the third and fifth week of June and the second week of July, respectively. The rates then decreased to 4.1% ± 2.5% from the fourth week of July and remained low from the second week of August to the third week of September. These trends were consistent throughout the 3-year study, and the parasitism rates in *M. saltuarius* exhibited a similar pattern to those in *M. alternatus* (Figure 2A,B).

From the fifth week of May to the fourth week of July, the parasitism rates of *C. flavator* were consistently higher than those of *S. verustus*. However, *S. verustus* exhibited increased parasitism rates during a period of statistically significant low rates for *C. flavator* in the fourth week of August (2.0% ± 0.1% in *M. alternatus* and 1.5% ± 0.5% in *M. saltuarius*) (Figure 2C,D). Despite the generally lower rates, *S. verustus* was consistently present in both *M. alternatus* and *M. saltuarius* throughout the study period.

## 4. Discussion

### 4.1. Development of the Vectors and Release Time of Parasitoids upon Field Application

To ensure the effective management of pest populations, it is crucial to determine the optimal release time of biological control agents, which is generally closely correlated with the life cycle of the host species. For this reason, we compared the developmental stages of *M. alternatus* and *M. saltuarius* with the seasonal occurrences of their parasitoids, *C. flavator,* and *S. verustus*, at the study sites (Figure 2).

*M. alternatus* and *M. saltuarius*, well-known vectors of pine wilt disease in South Korea, share similar hosts and life cycles. Kim et al. [25] reported that the activity period of adult *M. alternatus* extends from mid-May to early October, with a peak in June, and Han et al. [26] observed that the activity period for mature *M. saltuarius* runs from early May to August, peaking in late May. Therefore, the initial emergence of *M. saltuarius* occurs approximately 2 weeks earlier than that of *M. alternatus*.

The life cycles of *M. alternatus* and *M. saltuarius* exhibit significant similarities [38,39]. Both *M. alternatus* and *M. saltuarius* have a pre-oviposition period of approximately 2 weeks, and female adults have a lifespan of approximately 8 weeks [40,41,42,43,44]. Both species have an egg period of approximately 1 week. Upon hatching, larvae initially fed along the cambium beneath the bark for an average of 6 weeks before boring into the sapwood [24,44]. However, *M. saltuarius* larvae followed a distinct pattern, moving into the sapwood only after reaching the third instar. Additionally, *M. saltuarius* showed highly variable development durations, with the third instar lasting 30–130 d and the fourth instar ranging from 44–180 d. This variability makes it very challenging to accurately gauge the duration of the sub-bark period duration of vector larvae growing beneath the bark of their host trees [45].

**Figure 3:**
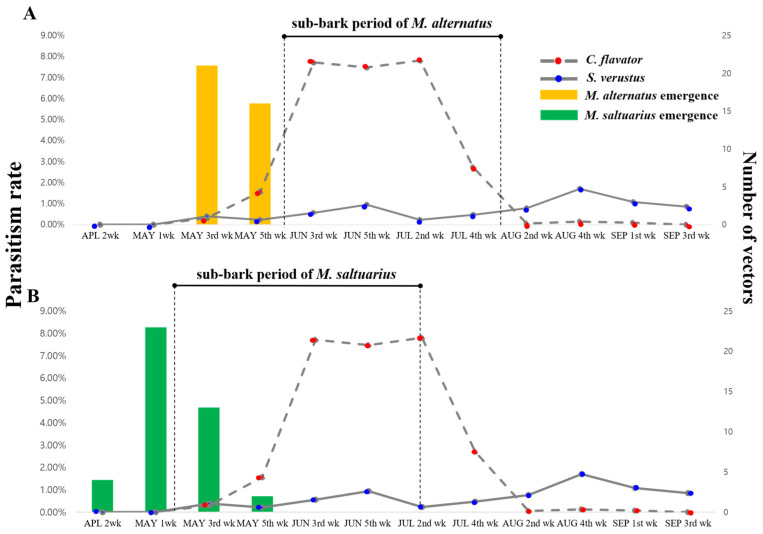
Parasitism rate fluctuation (on left axis) across the season by *C. flavator* and *S. verustus* with reference to host abundance (on right axis). (**A**) *C. flavator* and *S. verustus* on *M. alternatus*. (**B**) *C. flavator* and *S. verustus* on *M. saltuarius*. Number of captured adult *M. alternatus* (yellow bar) and *M. saltuarius* (green bar). (Sub-bark periods of both hosts = data exerted from [24,44].

The occurrence of *C. flavator*, which is nearly zero from the third to the fifth week of May, coincided with the pre-oviposition and egg incubation periods of *M. alternatus* [24]. Subsequently, the occurrence of *C. flavator* began to increase, reaching its peak from the third week of June to the second week of July, declining from the fourth week of July, and then becoming almost negligible by the second week of August onwards. The peak period of *C. flavator* occurrence (third week of June to the second week of July) aligns with the time when *M. alternatus* larvae live under the bark (early June to late July) [44] (Figure 3A). However, the occurrence of *C. flavator* did not show a strong correlation with that of *M. saltuarius* [24,38] as it did with that of *M. alternatus* (Figure 3B).

The occurrence of *S. verustus* did not exhibit a specific association with any particular developmental stage of *M. alternatus* or *M. saltuarius.* Its occurrence generally decreased, with an increase in the occurrence of *C. flavator* (in the fifth week of May), and increased when *C. flavator* was less frequent or nearly absent (by the second week of August) (Figure 3). However, since these parasitoids are known to parasitize many hosts [46,47,48] other than *M. alternatus* or *M. saltuarius*, it is hypothesized that *C. flavator* has a higher host preference for *M. alternatus* during the beetle’s developmental period rather than competition for a host. This aspect of host preference needs to be investigated in further research, testing the other host species if available.

Conclusively, *C. flavator* shows a higher host preference for *M. alternatus* over *M. saltuarius*. The occurrence of *C. flavator* is closely correlated with the availability period of *M. alternatus* larvae developing under the bark (Figure 3A). Such a close association was not observed between the occurrence of *C. flavator* and the presence of *M. alternatus* larvae [24,49] (Figure 3B). Nevertheless, since the occurrence of *M. saltuarius* largely coincides with that of *M. alternatus* and the parasitism rate of *C. flavator* is relatively high, it is anticipated that the release of *C. flavator* can also be effective during the larval period of *M. saltuarius* [25,26]. Furthermore, although *S. verustus* exhibited lower parasitism rates than *C. flavator*, it remained active from the second week of August to the third week of September and peaked in the fourth week of August.

With the finding above, we carefully suggest that the optimal release time for *C. flavator*, considering the activity periods of the two vectors, would be concentrated in June and July. Additionally, since *M. alternatus* can continue oviposition until mid-September [50], the release of *S. verustus* from August to late September may ensure continuous biological control of the extended parasitization period on these vectors. However, the optimal release time of both parasitoids must be evaluated through further research, prior to the initiation of a biological control program.

### 4.2. Effective Release Locations for C. flavator

**Figure 4:**
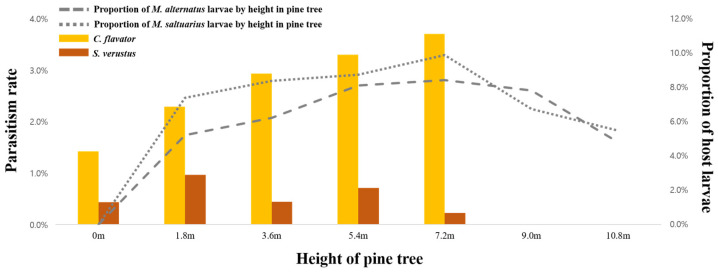
Parasitism rate (left axis) across heights of trees by *C. flavator* (yellow bar) and *S. verustus* (brown bar) with reference to host abundance (two dotted grey lines on the right axis = the data exerted from [22,51]).

According to on our survey results, focusing on the release height rather than the forest depth and *S. verustus* is advantageous when mass-releasing *C. flavator.* According to Futai et al. [22,51], *M. alternatus* larvae are sparsely distributed inside pine trees below 1.5 m and reach their highest density between 5.1 and 7.8 m. Our study confirmed that the parasitism rates increased with the installation height of sentinel logs and peaked at 7.2 m (Figure 4). Consequently, our survey suggests that the preferred parasitism height of *C. flavator* aligns with the distribution of *M. alternatus* larvae. Therefore, if *C. flavator* is released at a height of 5–7 m on pine trees, where the larval density of both vectors is the highest, it would be an effective strategy for biological control.

### 4.3. Sex Ratio of C. flavator

*C. flavator* exhibits a male-biased sex ratio in both *M. alternatus* (Table 3). Charnov et al. [52] observed that when hosts vary significantly in size, eggs laid on smaller hosts are more likely to develop into males, but those laid on larger hosts tend to become females. Furthermore, there is a tendency for more eggs to develop into males when host sizes are similar, indicating that host size influences sex determination in a relative manner rather than in an absolute manner. It is important to note that not all larval instars within the sentinel logs used in this survey were measured, but the induction of oviposition from the vectors for only 1 week suggests that the larval instars within the logs were relatively uniform. Furthermore, *t*-test results revealed no statistically significant differences in the head widths of *M. alternatus* larvae, from which male and female wasps emerged, across all instars. This suggests that the sizes of the host larvae chosen by the parasitoids are generally consistent, corroborating the findings of Charnov et al. [52] (Table 2 and Table 4).

A female-biased sex ratio in parasitoids is considered crucial for enhancing the efficacy of biological control [53]. Therefore, conducting additional experiments with hosts of various sizes, especially using larger larvae, could potentially help adjust the sex ratio of *C. flavator* to be more female-biased in mass rearing.

### 4.4. Host Size Preference of Parasitoids

The host–searching behavior of parasitoids, which is crucial for oviposition, typically demonstrates clear, purpose-driven preferences, particularly among those that feed on various host species. They meticulously select hosts to ensure successful oviposition, often favoring specific sizes within the same developmental stages of hosts [54,55,56]. In this experiment, *C. flavator* and *S. verustus* specifically targeted certain larval instars of *M. alternatus* and *M. saltuarius*. *C. flavator* predominantly parasitized the fourth instar of *M. alternatus* (51.7%) and the third instar of *M. saltuarius* (71.4%), but *S. verustus* preferred the fourth instar of *M. alternatus* (50.0%) and the second instar of *M. saltuarius* (54.5%) (Table 3). Although oviposition by the vectors was induced within each breeding box for only a week, with 10 female vectors laying eggs on three sentinel logs simultaneously, variations in light and developmental rates at log installation sites suggest that multiple larval instars were present within the sentinel logs. It was observed that *C. flavator* prefers larvae within a head width range of 1.52 to 2.4 mm, which corresponds to the fourth instar of *M. alternatus* and the third instar of *M. saltuarius*, and *S. verustus* prefers larvae within a head width range of 1.03 to 2.4 mm, which corresponds to the fourth instar of *M. alternatus* and the second instar of *M. saltuarius*. However, due to the uniform duration of induced oviposition, it is unclear whether the abundance of specific instars was coincidental or truly indicative of the preference of the parasitoids. This uncertainty necessitates further experiments on host instar preference under varied and controlled conditions.

### 4.5. Assessment of Natural Enemy Utilization

Research on natural enemies of pine wilt disease vectors has been conducted in South Korea by Kim et al. [57] and Jang et al. [17]. However, these studies encountered difficulties in accurately identifying the host trees and determining the parasitism rates of major parasitoids. Our field survey from 2018 to 2020 identified 15 species of parasitoids affecting both *M. alternatus* and *M. saltuarius*, with four species parasitizing both vectors (Table 1). Notably, *C. flavator* and *S. verustus* exhibited the highest parasitism rates and demonstrated a clear preference for *M. alternatus* over *M. saltuarius* (Table 2).

Despite the generally low parasitism rates of the larval parasitoids observed during the survey period (Table 2), the highest rates were recorded at the third site in July 2018. *C. flavator* achieved a parasitism rate of 26.1% in *M. alternatus* and 24.5% in *M. saltuarius*. *S. verustus* reached a parasitism rate of 6.1% in *M. alternatus* in June 2018 and 3.9% in *M. saltuarius* in August 2018 (Figure 2). These results showed that these parasitism rates were lower compared to natural enemies of the Emerald ash borer in China [58].

More comprehensive and diverse experiments are necessary to demonstrate their efficacy in utilizing these parasitoids as effective biological control agents. *C. flavator* also has been confirmed as a primary parasitoid against *M. galloprovincialis*, a vector of pine wilt disease in Portugal, but it achieved a parasitism rate of just about 10% on *M. galloprovincialis*, which would not be effective to reduce the population of the insect vector in Portugal [49]. *S. verustus* has once shown a maximum parasitism rate of 48.88% against *M. alternatus* in Jinju, Gyeongsangnam-do, Republic of Korea [18]. Such a high parasitism rate was not observed during the surveys of the present study. Although we cannot fathom a clear reason for this difference in the rates, in the current situation, parasitism rates may significantly vary due to various factors such as host availability, density, occurrence timing, location, and experimental conditions (indoor vs. outdoor).

Overall, the average parasitism rate of both parasitoids is less than 10%, which is not recommendable for biological control agents. Surely, *C. flavator* was able to achieve more than 20% of the rate, but only for a brief period of time. That may not lead to an actual reduction of the hosts and to slowing down the spread of the disease. There seems to be another obstacle to rearing *C. flavator* in mass for release. Although we have not found much about biological traits of *C. flavator*, one thing clear enough is that the wasp is solitary, laying only one egg per host. This fact may lead to very slow production of the wasp for a release and also requires more production cost, including a huge amount of wood blocks for host rearing. Considering these characteristics of *C. flavator*, the wasp does not appear to guarantee a successful mass-rearing for a biological control program. However, as mentioned above, we have not found all the biological traits of the wasp, such as fecundity, longevity, etc. Therefore, any conclusion should be made after an extensive study on their biology and life history in order to determine whether the wasp has enough potential as a biological control agent.

## 5. Conclusions

This study provides valuable insights into the utilization of natural enemies, specifically, the parasitoids *C. flavator* and *S. verustus*, in controlling the vectors of pine wilt disease, *M. alternatus* and *M. saltuarius*. The findings suggest that these parasitoids, *C. flavator* and *S. verustus*, exhibit distinct preferences and parasitism rates for their hosts, with *C. flavator* showing a higher efficacy against *M. alternatus*. By summing up all of the data regarding the hosts and parasitoids, the optimal release time for these parasitoids was inferred to be in June and July for *C. flavator* and from August to September for *S. verustus*. Additionally, it would be effective if these parasitoids are released at heights between 5 and 7 m on pine trees. Further research is needed to clarify the release strategies and evaluate the host size preferences of parasitoids under varied conditions to maximize their potential as biological control agents.

## Figures and Tables

**Table 1:** Parasitoids and their parasitism rates on *M. alternatus* and *M. saltuarius* larvae determined using sentinel logs at Pohang-si, Gyeongsangbuk-do, Republic of Korea, in 2018.

Host	Parasitoids	Total Parasitism Rate (Number of Parasitized Larvae/ Total Number of Host Larvae)
*M. alternatus* (Total number of larvae: 2715)	*Cyanopterus flavator*	5.8%
	*Spathius verustus*	1.3%
	*Doryctes strialellus*	0.2%
	*Rhaconotus formosanus*	0.1%
	*Xorides sepulchralis*	0.03%
	*Ichneumonidae* sp. 1	0.03%
	*Ichneumonidae* sp. 2	0.03%
*M. saltualius* (Total number of larvae: 4076)	*Cyanopterus flavator*	1.6%
	*Doryctes strialellus*	0.6%
	*Spathius verustus*	0.5%
	*Rhaconotus formosanus*	0.3%
	*Sclerodermus harmandi*	0.1%
	*Heydenia* sp. 1	0.1%
	*Heydenia* sp. 2	0.1%
	*Pteromalidae* sp. 1	0.1%
	*Pteromalidae* sp. 2	0.1%
	*Pteromalidae* sp. 3	0.1%
	*Pteromalidae* sp. 4	0.02%
	*Pteromalidae* sp. 5	0.02%

**Table 2:** Parasitism rates of *C. flavator* and *S. verustus* on *M. alternatus* and *M. saltuarius*.

Parasitoid (Parasitism Type)	Host	Total Parasitism Rate (*n*)	*p*-Value	Sex Ratio
*C. flavator* (solitary)	*M. alternatus*	6.3% (1350)	2.2 × 10^−16^ ***	0.8
	*M. saltuarius*	1.0% (1350)		0.9
	*M. alternatus* *+* *M. saltuarius*	3.4% (2700)	-	0.8
*S. verustus* (gregarious)	*M. alternatus*	0.7% (1350)	0.49	0.2
	*M. saltuarius*	0.5% (1350)		0.2
	*M. alternatus* *+* *M. saltuarius*	0.6% (2700)	-	0.2

*** *p* < 0.001.

**Table 3:** Parasitism rates of *C. flavator* and *S. verustus* on host larval instars of two *Monochamus* species.

Parasitoid	Host	Larval Instar (Range of Head Capsule Width, mm)	Mean ± SD Head Capsule Width (mm) with Sample Size n in Bracket (OR ± SE)	Parasitism Rate % *
*C. flavator*	*M. alternatus*	1st (0–1.1)	0.54 ± 0.02 (2)	1.7
		2nd (1.1–1.4)	1.03 ± 0.12 (4)	3.4
		3rd (1.4–1.9)	1.39 ± 0.15 (15)	12.7
		4th (1.9–2.4)	2.24 ± 0.11 (61)	51.7
		5th (2.4–2.8)	1.11 ± 0.07 (25)	21.2
		8th (3.3–3.6)	3.75 ± 0.18 (11)	9.3
	*M. saltuarius*	1st (0.64–1.00)	1.11 ± 0.17 (2)	4.1
		2nd (1.03–1.5)	1.75 ± 0.14 (28)	57.1
		3rd (1.52–2.3)	2.19 ± 0.22 (12)	24.5
		4th (2.31–3.81)	2.85 ± 0.07 (7)	14.3
*S. verustus*	*M. alternatus*	2nd (1.1–1.4)	1.11 ± 0.01 (2)	7.1
		3rd (1.4–1.9)	1.14 ± 0.1 (7)	25.0
		4th (1.9–2.4)	2.22 ± 0.12 (14)	50.0
		5th (2.4–2.8)	2.55 ± 0.11 (5)	17.9
	*M. saltuarius*	1st (0.64–1.00)	1.13 ± 0.12 (13)	59.1
		2nd (1.03–1.5)	1.72 ± 0.12 (8)	36.4
		3rd (1.52–2.3)	1.99 ± 0.00 (1)	4.5

* (number of parasitized host larvae in each instar/total number of larvae) × 100.

**Table 4:** A comparison in the head capsule between host larvae at varying instars yielding male and female offspring of *C. flavator*.

*M. alternatus*		Sex of *C. flavator* (*n*)	*t*-Test
Larval Instar	Average Head Capsule Width ± SD (mm)		
1st	1.22 ± 0.18	Female (2)	0.09
	0.93 ± 0.11	Male (4)	
2nd	1.45 ± 0.04	Female (2)	0.12
	1.34 ± 0.09	Male (30)	
3rd	1.73 ± 0.33	Female (2)	0.08
	1.7 ± 0.18	Male (16)	
4th	2.36 ± 0.18	Female (41)	0.09
	2.31 ± 0.15	Male (156)	
5th	3.69 ± 0.2	Female (9)	0.06
	3.66 ± 0.21	Male (19)	

**Table 5:** Parasitism rate by *C. flavator* and *S. verustus* of host larvae regarding height of sentinel logs and forest depth.

Parasitoid	Variable	Interaction Effect Between Height and Depth (*p*-Value)	Distance (m) from the Ground on Trunks/from the Edge in Forest	Total Parasitism Rate †	*p*-Value
*C. flavator*	Height of sentinel log	0.06	0	1.7% ^a^	2.2 × 10^−16^ ***
			1.8	2.6% ^ab^	
			3.6	3.5% ^bc^	
			5.4	4.2% ^bc^	
			7.2	5.2% ^c^	
	Forest depth		0	3.4%	0.65
			20	3.6%	
			40	3.3%	
*S. verustus*	Height of sentinel log	0.12	0	0.4% ^ab^	0.003 **
			1.8	1.0% ^c^	
			3.6	0.4% ^ab^	
			5.4	0.7% ^b^	
			7.2	0.2% ^a^	
	Forest depth		0	0.6%	0.55
			20	0.5%	
			40	0.6%	

† Means with alphabetical letters (a–c) within a column indicate significant difference (*p* < 0.05) among heights of sentinel logs and forest depths (post hoc tests by Tukey HSD). *** *p* < 0.001, ** *p* < 0.01.

## Data Availability

The data presented in this study are available on request from the corresponding author. The data are not publicly available due to “the regulation of research management” of the Korea National Arboretum.

## Supplementary Materials

The following supporting information can be downloaded at: https://www.mdpi.com/article/10.3390/insects15120943/s1, Figure S1. Parasitoids of *M. alternatus* and *M. saltuarius* (Bracondiae: A–D, Bethylidae: E, Ichneumonidae: F–H, Pteromalidae: I–O). (A) *Cyanopterus flavator*; (B) *Spathius verustus*; (C) *Doryctes striatellus*; (D) *Rhaconotus formosanus*; (E) *Sclerodermus harmandi*; (F) *Xorides sepulchralis*; (G) Ichneumonidae sp. 1; (H) Ichneumonidae sp. 2; (I) *Heydenia* sp. 1; (J) *Heydenia* sp. 2; (K) Pteromalidae sp. 1; (L) Pteromalidae sp. 2; (M) Pteromalidae sp. 3; (N) Pteromalidae sp. 4; (O) Pteromalidae sp. 5.
