# Supplementary material for: Ecological and Biological Studies of Two Larval Parasitoids on Two Monochamus Vectors of the Pinewood Nematode in South Korea
Source: Insects. 2024 Nov 29;15(12):943. doi: 10.3390/insects15120943 (PMC11678335; doi:10.3390/insects15120943)
Supplement: Supplementary file 1 [file insects-15-00943-s001.zip › insects-3272061-supplementary.pdf]

Supplementary Materials

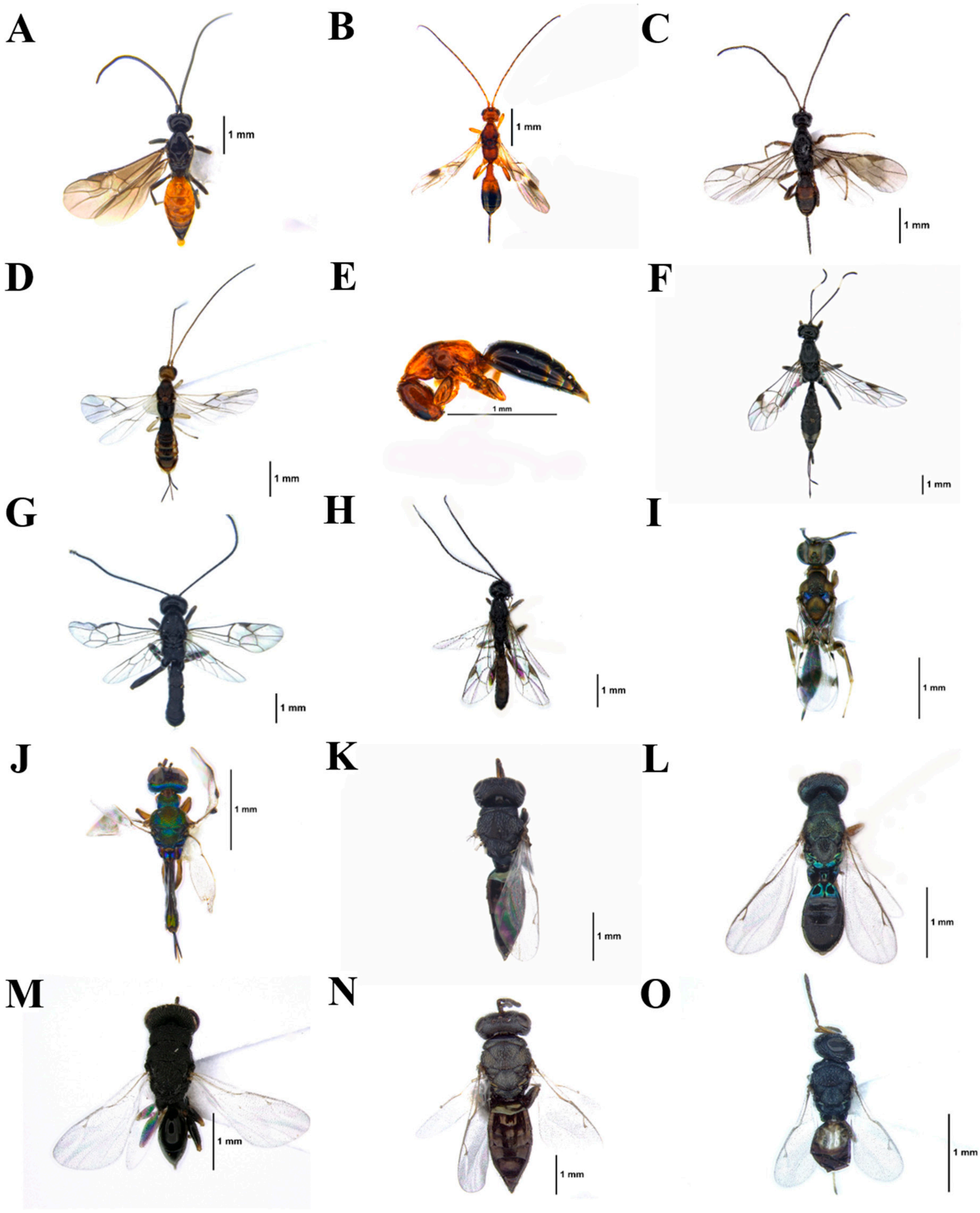

Supplemental Figure S1. Parasitoids of *M. alternatus* and *M. saltuarius* (Bracondiae: A–D, Bethylidae: E, Ichneumonidae: F–H, Pteromalidae: I–O).

(A) *Cyanopterus flavator*; (B) *Spathius verustus*; (B) *Doryctes striatellus*; (D) *Rhaconotus formosanus*; (E) *Sclerodermus harmandi*; (F) *Xorides sepulchralis*; (G) Ichneumonidae sp. 1; (H) Ichneumonidae sp. 2; (I) *Heydenia* sp. 1; (J) *Heydenia* sp. 2; (K) Pteromalidae sp. 1; (L) Pteromalidae sp. 2; (M) Pteromalidae sp. 3; (N) Pteromalidae sp. 4; (O) Pteromalidae sp. 5.
